# Supplementary material for: Measuring luteinising hormone pulsatility with a robotic aptamer-enabled electrochemical reader
Source: Nat Commun. 2019 Feb 20;10:852. doi: 10.1038/s41467-019-08799-6 (PMC6382769; doi:10.1038/s41467-019-08799-6)
Supplement: Supplementary file 1 — Supplementary Information [file 41467_2019_8799_MOESM1_ESM.pdf]

Measuring luteinising hormone pulsatility with a robotic aptamer-enabled electrochemical reader

Liang et al.

Supplementary Information

Measuring luteinising hormone pulsatility with a robotic aptamer-enabled electrochemical reader

Shaolin Liang[1,2,3], Andrew B Kinghorn[1], Margaritis Voliotis[4], Julia K. Prague[2], Johannes D. Veldhuis[5], Krasimira Tsaneva-Atanasova[4], Craig A. McArdle[6], Raymond H.W. Li[7], Anthony E.G. Cass[3,*], Waljit S. Dhillo[2,*] & Julian A. Tanner[1,*]

[1]School of Biomedical Sciences, LKS Faculty of Medicine, The University of Hong Kong, Hong Kong, China.
[2]Section of Endocrinology and Investigative Medicine, Imperial College London, UK.
[3]Department of Chemistry, Imperial College London, London, UK.
[4]Department of Mathematics and Living Systems Institute, College of Engineering, Mathematics, and Physical Sciences, University of Exeter, Exeter, UK.
[5]Endocrine Research Unit, Mayo School of Graduate Medical Education, Mayo Clinic, Minnesota, USA.
[6]Laboratories for Integrative Neuroscience and Endocrinology, School of Clinical Sciences, University of Bristol, Bristol, UK.
[7]Department of Obstetrics and Gynaecology, LKS Faculty of Medicine, The University of Hong Kong, Hong Kong, China.
*e-mail: t.cass@imperial.ac.uk; w.dhillo@imperial.ac.uk; jatanner@hku.hk

Supplementary Table 1 | Primer Sequences

N35 library:<br>5' – CGTACGGTCGACGCTAGC – N35 – CACGTGGAGCTCGGATCC – 3'
Forward primer for the amplification step of SELEX:<br>5' – CGTACGGTCGACGCTAGC – 3'
Biotinylated Reverse primer for the amplification step of SELEX:<br>5' – biotin – GGATCCGAGCTCCACGTG – 3'
Biotinylated complementary strand for the 5'end primer region for ELONA assay:<br>5' – GCTAGCGTCGACCGTACG – 3'

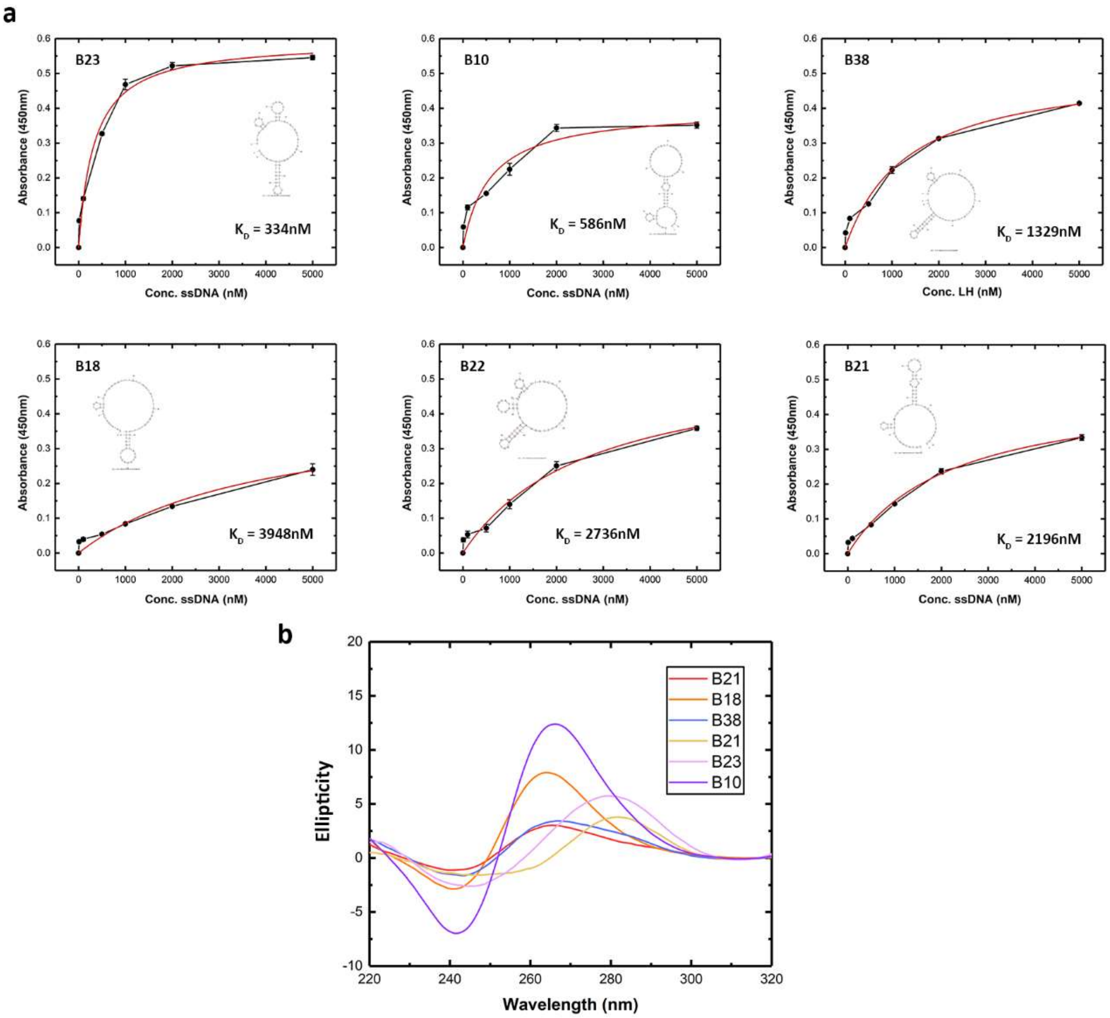

Supplementary Fig. 1 | ELONA of all six aptamer candidates. a, ELONA study was performed to initially screen aptamer candidate based on the affinity. B23 and B10 shows the best binding performance, estimated K_D = 334 nM and 586 nM, seperately. The Mfold prediction of the secondary structures is shown on each figure. **b,** CD spectra result of the six aptamer candidates. B10 and B18 share similar G-quadruplex structures and B23 shows an alternate G-quadruplex structure. No obvious G-quadruplex structures were observed from other candidates. Source data are provided as a Source Data file.

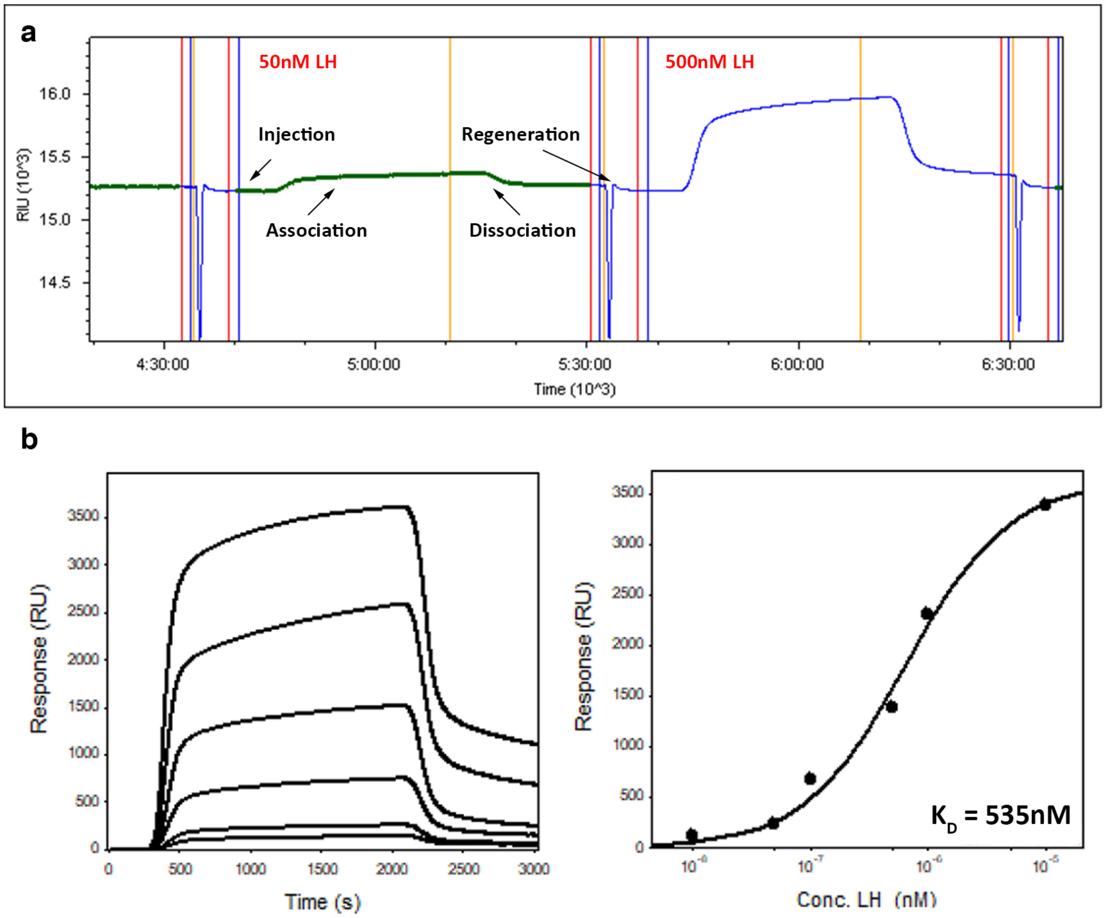

Supplementary Fig. 2 | Surface Plasmon Resonance (SPR) study to investigate the binding kinetics of B23. a, Injection-association-dissociation-regeneration cycles of the SPR study. Different stages are pointed out by the black arrows and the figure includes two concentrations, one is the response of the B23 aptamer against 50 nM LH and the other is response of 500 nM LH. **b,** SPR response of the B23 aptamer against different concentrations of LH from 10 nM to 10000 nM and the affinity estimation from the scrubber programme. K_D is estimated to be 535 nM.

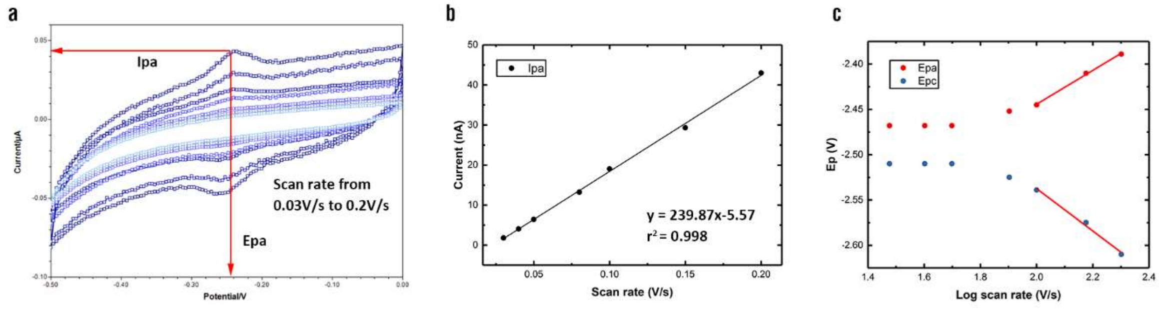

Supplementary Fig. 3 | Detailed electrochemical characterisation of the LH aptamer electrode. **a,** Cyclic Voltammogram for MB-modified LH aptamer immobilised electrode in PBS buffer under scan rates (0.03, 0.04, 0.05, 0.08, 0.1, 0.15 and 0.2V/s). The red arrows point out the oxidation peak current (Ipa) and oxidation peak potential (Epa). **b,** Plot Ipa vs. Scan rate. Linear relationship indicates the electron transfer is under a surface-controlled mechanism. **c,** Plot Ipa vs. log v. At high scan rates, both Epa and Epc exhibit linear relationship vs. log v. Source data are provided as a Source Data file.

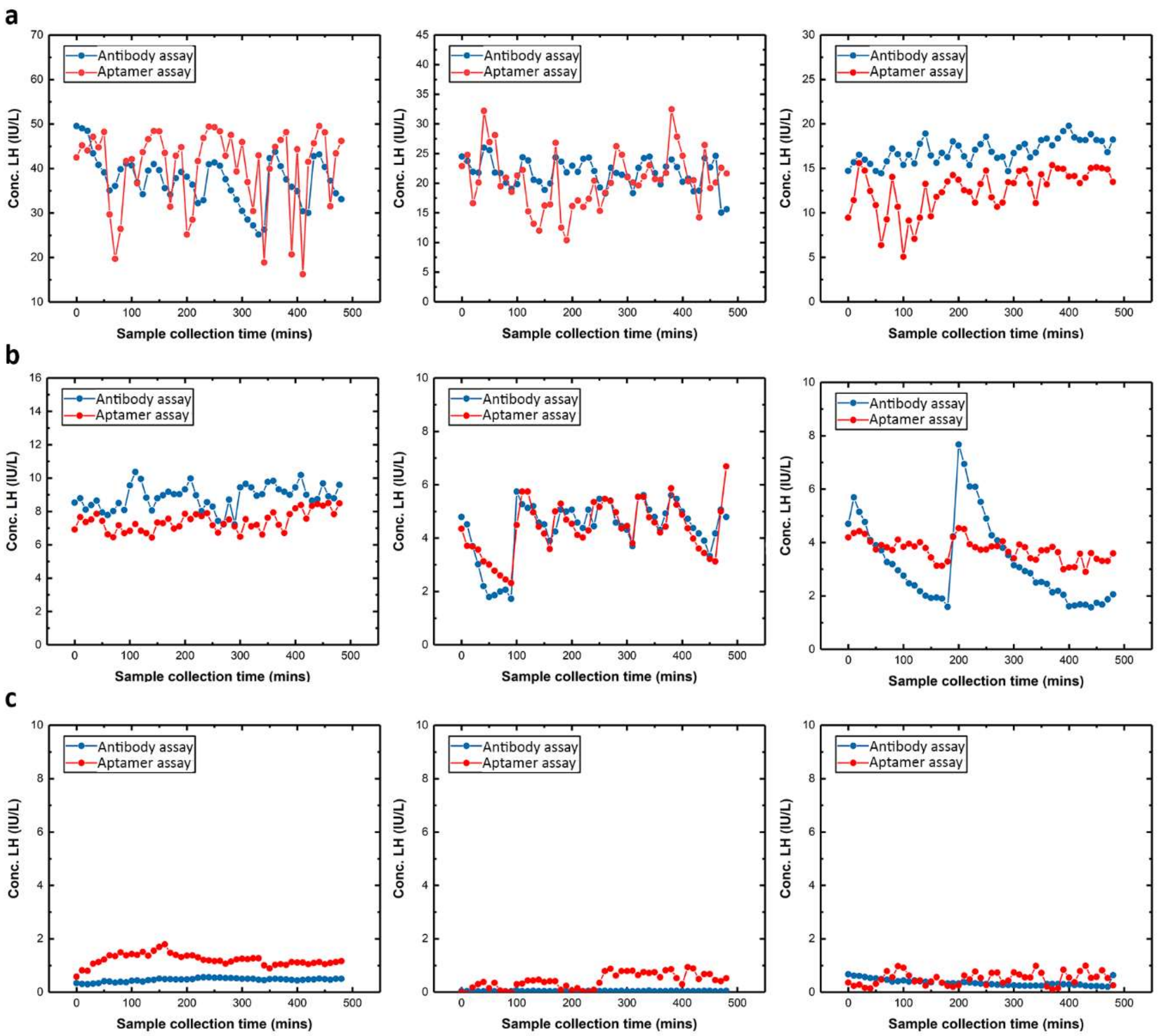

Supplementary Fig. 4 | LH pulsatility profiles of all patients measured both by clinical assay and aptamer assay. a, Menopause female (n=3) samples taken every 10 minutes in 8 hours by frequent sampling. **b,** Healthy female (n=3) samples taken every 10 minutes in 8 hours by frequent sampling. **c,** Women with hypothalamic amenorrhea (n=3) samples taken every 10 minutes in 8 hours by frequent sampling. Source data are provided as a Source Data file.

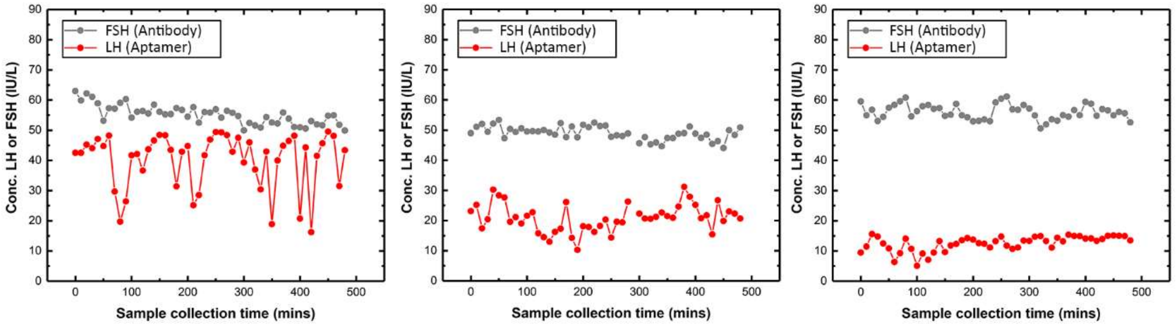

Supplementary Fig. 5 | LH pulsatility profile measured by aptamer assay and FSH profile measured by clinical assay of menopause patients. No significant correlation between two values. The aptamer assay shows strong specificity against LH. Source data are provided as a Source Data file.
